# Supplementary material for: Thermal management and non-reciprocal control of phonon flow via optomechanics
Source: Nat Commun. 2018 Mar 23;9:1207. doi: 10.1038/s41467-018-03624-y (PMC5865216; doi:10.1038/s41467-018-03624-y)
Supplement: Supplementary file 1 — Supplementary Information(PDF 1385 kb) [file 41467_2018_3624_MOESM1_ESM.pdf]

Supplementary information for "Thermal management and non-reciprocal control of phonon flow via optomechanics"

Seif *et al.*

Supplementary Note 1. TRANSMISSION AND REFLECTION COEFFICIENTS

To study transmission properties of the system, we consider the case where the beam is connected to two contacts at both ends. The Hamiltonian describing the optomechanical system is given by

$$\hat{H}_{\rm eff} = -\Delta/2\sum_n \hat{a}_n^\dagger \hat{a}_n + \omega_{\rm mech}/2\sum_n \hat{b}_n^\dagger \hat{b}_n - J\sum_n \hat{a}_{n+1}^\dagger \hat{a}_n - t\sum_n \hat{b}_{n+1}^\dagger \hat{b}_n - G\sum_n e^{-in\theta}\hat{a}_n^\dagger \hat{b}_n + \text{h.c.}, \tag{1}$$

where $\hat{a}_n(\hat{b}_n)$ is a bosonic operator that destroys a photonic (phononic) excitation at site n, and G is the enhanced optomechanical coupling rate. The detuning Δ is taken to be very close to the mechanical energy $\omega_{\rm mech}$. The parameters J and t denote the hopping strength of photons and phonons in adjacent cavities, respectively. We assume that the contacts are identical to the system. The coupling $Ge^{i\theta n}$ is turned "on" for the system in sites $0 \le n < N$, and is "off" in the contacts (sites $n < 0$, and $n \ge N$).

In order to study the transmission properties of our system, we begin by considering phonons with incoming amplitude $e^{ik_{\rm b}n}$ incident from the left. As shown in Supplementary Figure 1, this gives rise to amplitudes $R_{\rm b}e^{-ik_{\rm b}n}$ and $T_{\rm b}e^{ik_{\rm b}n}$ for the reflection and transmission of phonons, respectively. The optomechanical coupling in $\hat{H}_{\rm eff}$ (Supplementary Equation 1) allows for conversion of phonons to photons. The amplitudes $R_{\rm a}e^{-ik_{\rm a}n}$ and $T_{\rm a}e^{ik_{\rm a}n}$ represent the processes in which the converted photons travel to the left and right, respectively. The parameters $k_{\rm a}$ and $k_{\rm b}$ are the wavenumbers of photons and phonons in the contacts.

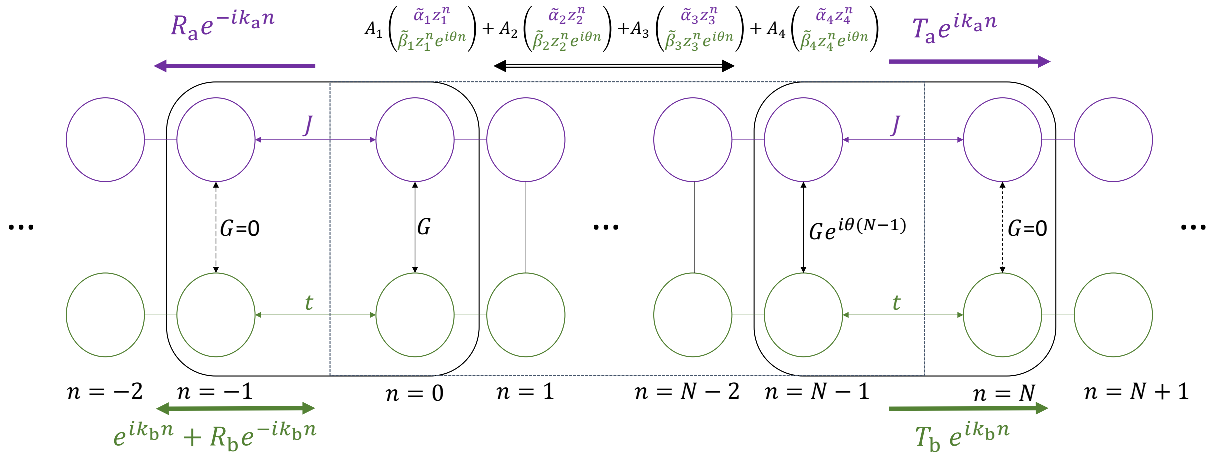

Supplementary Figure 1. **Schematic of transmission properties of the system.** The system is connected to two contacts at its boundaries (shown with dashed lines). At each site, n, in the system, phonons (green) are coupled optomechanically to photons (purple) with the strength $Ge^{i\theta n}$, where θ is an angle. Excitations hop to neighboring sites in the same leg of the ladder with strength t and J for phonons and photons, respectively. The wave function of photons and phonons in the system, can be expanded in terms of coefficients A_j, z_j, $\tilde{\alpha}_j$, and $\tilde{\beta}_j$. Contacts are identical to the system with the exception that G is set to zero. Excitations in the contacts can be expressed as plane waves $e^{\pm ik_{\rm a}n}$ for photons in the upper leg, and as $e^{\pm ik_{\rm b}n}$ for phonons in the lower leg, where $k_{\rm a(b)}$ is the wavenumbers associated with the dispersion of photons(phonons) in the contacts. When phonon waves enter the system from the left at site $n = 0$ and leave the system from site $n = N-1$, their transmission and reflection amplitudes are denoted by $T_{\rm b}$ and $R_{\rm b}$. The amplitudes $R_{\rm a}e^{-ik_{\rm a}n}$ and $T_{\rm a}e^{ik_{\rm a}n}$ represent the processes in which the converted photons travel to the left and right, respectively.

The amplitudes $R_{\rm a}$, $R_{\rm b}$,$T_{\rm a}$, and $T_{\rm b}$ can be found using the equations of motion of $\hat{H}_{\rm eff}$ (Supplementary Equation

1) at the two boundaries. In frequency space the equations give

$$\omega R_{\rm a} e^{ik_{\rm a}} = -J(R_{\rm a} e^{2ik_{\rm a}} + \alpha_0) \tag{2}$$
$$\omega(e^{-ik_{\rm b}} + R_{\rm b} e^{ik_{\rm b}}) = -t(e^{-2ik_{\rm b}} + R_{\rm b} e^{2ik_{\rm b}} + \beta_0) \tag{3}$$
$$\omega\alpha_0 = -J(R_{\rm a} e^{ik_{\rm a}} + \alpha_1) - G\beta_0 \tag{4}$$
$$\omega\beta_0 = -t(e^{-ik_{\rm b}} + R_{\rm b} e^{ik_{\rm b}} + \beta_1) - G\alpha_0 \tag{5}$$
$$\omega\alpha_{N-1} = -J(\alpha_{N-2} + T_{\rm a} e^{ik_{\rm a}N}) - Ge^{-i(N-1)\theta}\beta_{N-1} \tag{6}$$
$$\omega\beta_{N-1} = -t(\beta_{N-2} + T_{\rm b} e^{ik_{\rm b}N}) - Ge^{i(N-1)\theta}\alpha_{N-1} \tag{7}$$
$$\omega T_{\rm a} e^{ik_{\rm a}N} = -J(\alpha_{N-1} + T_{\rm a} e^{ik_{\rm a}(N+1)}) \tag{8}$$
$$\omega T_{\rm b} e^{ik_{\rm b}N} = -t(\beta_{N-1} + T_{\rm b} e^{ik_{\rm b}(N+1)}). \tag{9}$$

The parameters α_n and β_n represent the amplitudes of photon and phonon excitations in the system and can be expressed as

$$\begin{pmatrix} \alpha_n \\ \beta_n \end{pmatrix} = A_1 \begin{pmatrix} \tilde{\alpha}_1 z_1^n \\ \tilde{\beta}_1 z_1^n e^{i\theta n} \end{pmatrix} + A_2 \begin{pmatrix} \tilde{\alpha}_2 z_2^n \\ \tilde{\beta}_2 z_2^n e^{i\theta n} \end{pmatrix} + A_3 \begin{pmatrix} \tilde{\alpha}_3 z_3^n \\ \tilde{\beta}_3 z_3^n e^{i\theta n} \end{pmatrix} + A_4 \begin{pmatrix} \tilde{\alpha}_4 z_4^n \\ \tilde{\beta}_4 z_4^n e^{i\theta n} \end{pmatrix}, \tag{10}$$

and z_j, and $\begin{pmatrix} \tilde{\alpha}_j \\ \tilde{\beta}_j \end{pmatrix}$ are eigenvalues and eigenvectors of the Hamiltonian corresponding to four available modes in the system. As it can be seen, (Supplementary Equation 2)-(Supplementary Equation 9) form a closed set of equations, which can be solved for $R_{\rm a}$, $R_{\rm b}$, $T_{\rm a}$, and $T_{\rm b}$. We define reflection and transmission probabilities as

$$\mathcal{R}_{\rm a} = |R_{\rm a}|^2 \frac{v_{\rm a}}{v_{\rm b}}, \tag{11}$$
$$\mathcal{R}_{\rm b} = |R_{\rm b}|^2 \frac{v_{\rm b}}{v_{\rm b}} = |R_{\rm b}|^2, \tag{12}$$
$$\mathcal{T}_{\rm a} = |T_{\rm a}|^2 \frac{v_{\rm a}}{v_{\rm b}}, \tag{13}$$
$$\mathcal{T}_{\rm b} = |T_{\rm b}|^2 \frac{v_{\rm b}}{v_{\rm b}} = |T_{\rm b}|^2, \tag{14}$$

which satisfy the conservation of probability current

$$\mathcal{R}_{\rm a} + \mathcal{R}_{\rm b} + \mathcal{T}_{\rm a} + \mathcal{T}_{\rm b} = 1. \tag{15}$$

The band structure and the transmission probabilities of phonons as a function of their energy are shown in Supplementary Figure 2 and Supplementary Figure 3, respectively.

Supplementary Note 2. BAND STRUCTURE OF THE SUPERLATTICE

We calculate the phonon band structure for a beam with a unit cell as depicted in Supplementary Figure 4(a) using the finite-element (FEM) simulation package COMSOL. This band structure is used in calculation of current in the main text (see Supplementary Figure 4(b)). Repeating this nominal cell and deforming it in a periodic way (see Figure 3 in the main text) leads to the creation of a band of phonons that is composed of a coherent superposition of localized phononic states. As such, they strongly couple to a co-localized electromagnetic field. The localization of these modes means that they are well described by the tight-binding Hamiltonian $\hat{H}_{\rm eff}$ (Supplementary Equation 1). We take a particular deformation of the unit-cell (as shown in Figure 3 in the main text), and look at the bands close to the localized modes to verify our tight-binding approximations. In Supplementary Figure 5 we see a very good agreement between the simulation and our theoretical model.

Supplementary Note 3. CONTRAST CALCULATION AND ROOM TEMPERATURE RESULTS

As discussed in the main text we use a hybrid method to evaluate the contrast C. The calculations are done with the band-structure of the superlattice for frequencies below 25 GHz. In frequency range of 25 GHz to 3 THz a linear

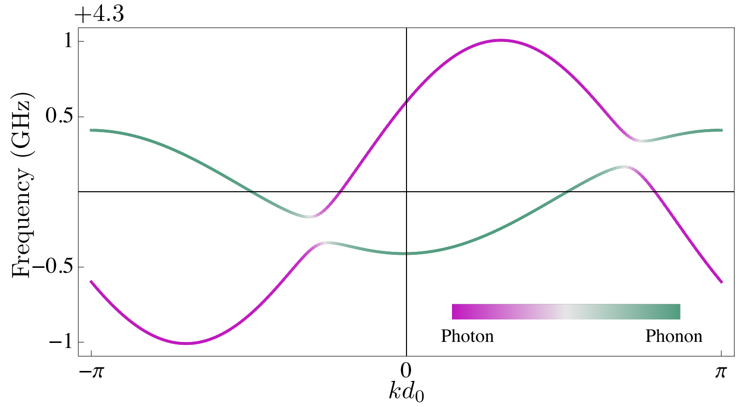

Supplementary Figure 2. **The band structure of a chain of coupled optomechanical cavities.** The band structure is shown for $\theta = 1.3\pi$ and parameters $\omega_{\rm mech}/2\pi = 4.3$ GHz, $J/2\pi = 0.5$ GHz, $t/2\pi = 0.2$ GHz, and $G/2\pi = 0.1$ GHz. Here, k is the wavenumber and d_0 is the lattice constant of the coupled optomechanical cavities. The gap is asymmetric under $k \to -k$ which results in non-reciprocal transport. The parameter θ controls the relative position of the gap, while G controls its width.

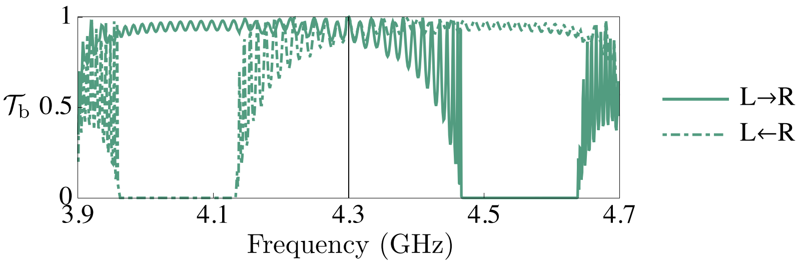

Supplementary Figure 3. **Transmission probabilities as a function of energy for a system with 100 sites**. The system is connected to two contacts and transmission probability $\mathcal{T}_{\rm b}$ is plotted for $\theta = 1.3\pi$ and parameters $\omega_{\rm mech}/2\pi = 4.3$ GHz, $J/2\pi = 0.5$ GHz, $t/2\pi = 0.2$ GHz, and $G/2\pi = 0.1$ GHz. In the case where phonon traveling from the left contact to the right contact (solid line) the gap is in the higher energies, compared to the transport in the right to left direction(dashed line). The transmission probability is close to zero in the gap.

dispersion ($\omega = v_{\rm s}k$) is assumed [1], and only at room temperature, where frequencies above 3 THz play an important role, mean free paths and band-structure from first-principle calculations are used and taken from Ref. [2]. We employ the Landauer formalism to calculate the current [3]. The total current is given by considering these contributions together with the current through the non-reciprocal tight-binding band

$$\begin{aligned} I(\Theta_{\rm L},\Theta_{\rm R}) = &\int_0^{\omega_{\rm SL}} \frac{d\omega}{2\pi}\hbar\omega M_{\rm SL}(\omega)\frac{\lambda_{\rm ph}}{\lambda_{\rm ph}+L_{\rm s}}[n_{\rm B}(\Theta_{\rm L},\omega)-n_{\rm B}(\Theta_{\rm R},\omega)] \\ &+\int_{\omega_{\rm SL}}^{\omega_c} \frac{d\omega}{2\pi}\hbar\omega f(\phi) M_{\rm bulk}(\omega)\frac{\lambda_{\rm ph}}{\lambda_{\rm ph}+L_{\rm s}}[n_{\rm B}(\Theta_{\rm L},\omega)-n_{\rm B}(\Theta_{\rm R},\omega)] \\ &+\int_{\omega_{\rm mech}-2t}^{\omega_{\rm mech}+2t} \frac{d\omega}{2\pi}\hbar\omega\frac{\lambda_{\rm ph}}{\lambda_{\rm ph}+L_{\rm s}}[\mathcal{T}_{\rm L\to R}n_{\rm B}(\Theta_{\rm L},\omega)-\mathcal{T}_{\rm L\leftarrow R}n_{\rm B}(\Theta_{\rm R},\omega)], \end{aligned} \quad (16)$$

where density of modes $M_{\rm SL}$ is calculated from the superlattice band structure. The density of modes in the bulk material, $M_{\rm bulk}$, is obtained from the linear dispersion (up to 3 THz) and first-principles calculations(above 3 THz). The last line of (Supplementary Equation 16) takes the effect of non-reciprocal band into account. The transmission probability of this band is the product of the probabilities $\mathcal{T}_{\rm L\rightleftarrows R}$ shown in Supplementary Figure 3 and the factor $\frac{\lambda_{\rm ph}}{\lambda_{\rm ph}+L_{\rm s}}$ that is calculated using the scattering rates discussed in the main text. The ratio of the non-reciprocal part

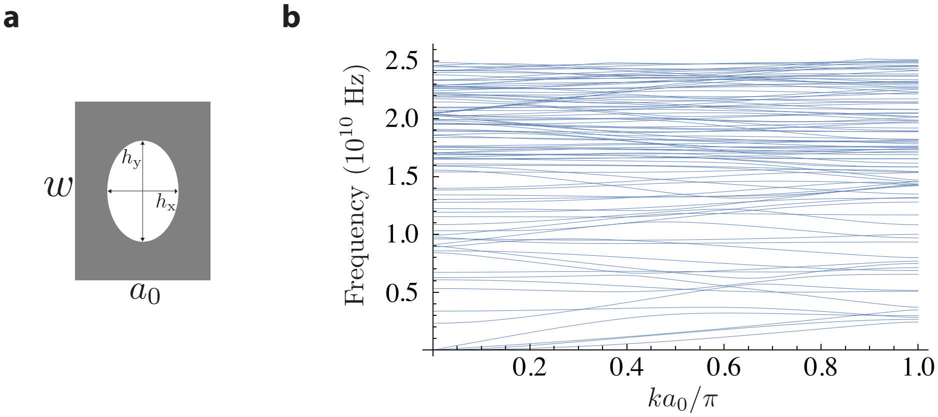

Supplementary Figure 4. **The nominal cell and its dispersion**. **a,** The nominal cell of the superlattice with $(w, a_0, h_x, h_y) = (600, 456, 240, 340)$ nm, and the thickness is 220 nm. **b,** band structure corresponding to this unit cell. We find that the band obeys the dispersion relation of the form $\omega = \omega_{\rm mech} + 2t\cos(kd_0x)$ with $\omega_{\rm mech} = 4.35651 \times 2\pi$ GHz, and $2t = 0.228 \times 2\pi$ MHz.

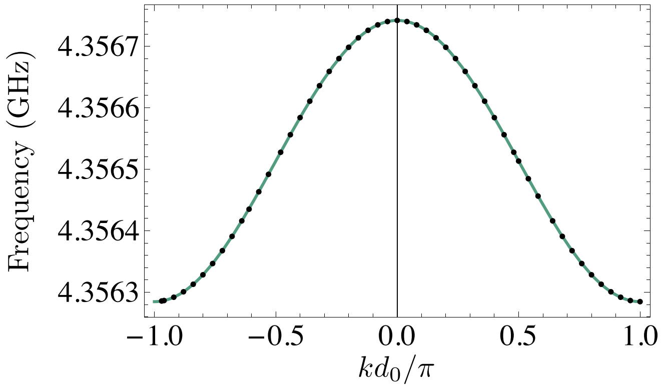

Supplementary Figure 5. **The dispersion of the phonons in an optomechanical cavity array**. The solid line is the prediction of tigh-binding model, and the dots are finite element simulation results. Here, k is the wavenumber and d_0 is the length of the unit cell. As we can see there is a great agreement between the theory and simulation. We find the dispersion to be $\omega = \omega_{\rm mech} + 2t\cos(kd_0x)$ with $\omega_{\rm mech} = 4.3565$ GHz, and $2t = 0.2$ MHz.

to the total current reported in the main text is given by

$$r(\Theta_{\rm L}, \Theta_{\rm R}) = \frac{\int_{\omega_{\rm mech}-2t}^{\omega_{\rm mech}+2t} \frac{d\omega}{2\pi} \hbar\omega \frac{\lambda_{\rm ph}}{\lambda_{\rm ph}+L_s} [\mathcal{T}_{\rm L\to R} n_{\rm B}(\Theta_{\rm L}, \omega) - \mathcal{T}_{\rm L\leftarrow R} n_{\rm B}(\Theta_{\rm R}, \omega)])}{I(\Theta_{\rm L}, \Theta_{\rm R})}. \tag{17}$$

The validity of the hybrid method rests on the separation of length scales of the problem. As seen in Supplementary Table 1, the length scale of each frequency interval is comparable to either the size of the unit-cell of the superlattice, or the size of the unit cell of the crystal, which corresponds to the dispersion that is used. In the main text we used

Frequency	Dispersion	Wavelength (unit-cell)
0-25 GHz	Superlattice band structure	200 nm (456 nm)
25 GHz - 3 THz	Linear dispersion (Debye)	200 nm - 2 nm
3 THz - 15 THz	First-principles	2 nm - 0.4 nm (0.5 nm)

Supplementary Table 1. Phonon frequencies and their corresponding length scale

the hybrid method to calculate the thermal current at 4 K and 0.4 K. Here, we use the same method to show the effect of impurities on the thermal conductivity of silicon at room temperature (see Supplementary Figure 6(a)) and calculate the contrast in $Si_{90}Ge_{10}$ optomechanical crystal with 10 nm nano-particles (see Supplementary Figure 6(b)).

The thermal conductivity is calculated using the Debye formula following Ref. [1]

$$\kappa = \int_0^{\omega_{\rm cut}} d\omega \frac{\hbar\omega}{2\pi}\frac{dn_{\rm B}}{dT}\tau(\omega)\frac{1}{2\pi}\omega^2\sum_{i=1}^{3}\frac{1}{c_i} \tag{18}$$

where the cutoff $\omega_{\rm cut}$ is taken to be 3 THz, and c_i's are the sound velocities of longitudinal and transverse branches. We use first-principle calculations [2] modified with Matthiessen rule for higher frequencies.In Supplementary Figure 6(a) we show the contrast C that is calculated using the described method.

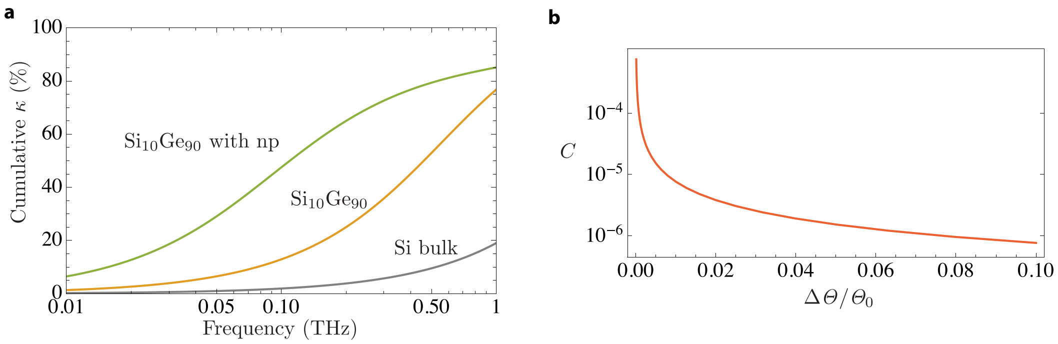

Supplementary Figure 6. **Thermal conductivity and contrast at room temperature**. **a**, Cumulative thermal conductivity (κ) of bulk silicon (gray), $\mathrm{Si_{90}Ge_{10}}$ (yellow), and $\mathrm{Si_{90}Ge_{10}}$ with nano-particles (green) as a function of phonon frequency at room temperature. **b**, Contrast C at room temperature versus the normalized temperature bias $\Delta\Theta/\Theta_0$ for the same optomechanical parameters used in the main text. In the limit of $\Delta\Theta \to 0$, the contrast approaches unity.

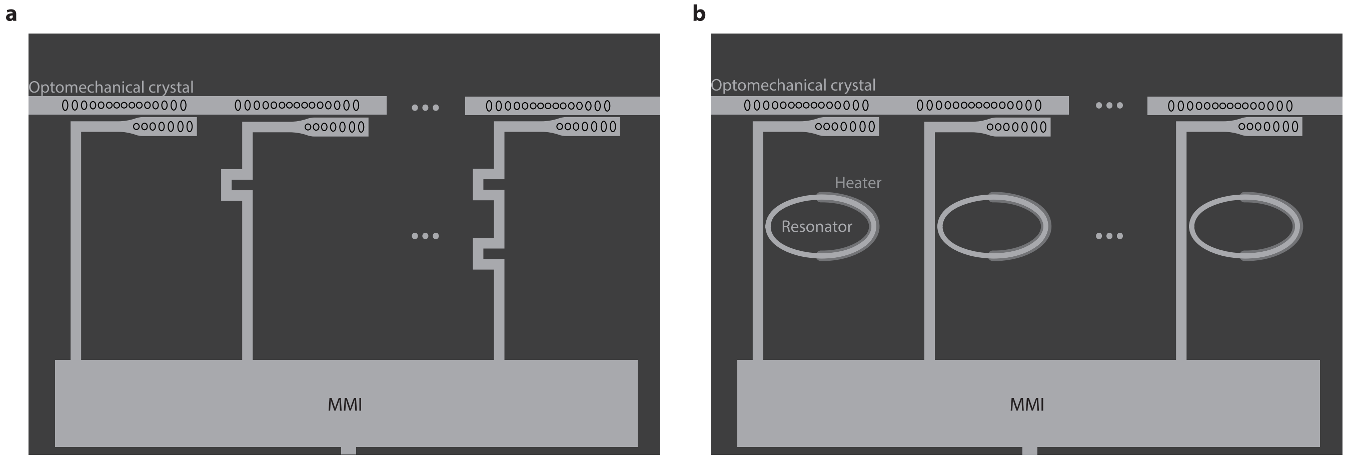

Supplementary Figure 7. **Schematic picture of the implementation of the phase gradient**. By using a multi-mode interference (MMI) beam splitter the power is distributed evenly to each waveguide. Each optomechanical cavity is evanescently coupled to a waveguide. **a**, By meandering the connections or **b**, by using heated zero-loss resonators as all-pass filters the propagation phase of the laser can be tuned.

Supplementary Note 4. ON-CHIP IMPLEMENTATION OF THE PHASE GRADIENT

An on-chip approach to implementing a position dependent phase in the laser drive, i.e. $\sum_n e^{i\theta n}\epsilon_{\rm d}\cos(\omega_{\rm d}t)(a_n+a_n^\dagger)$, is using the propagation phase of light ($\Delta\theta \propto e^{ik\Delta l}$) in a waveguide. We propose using a power divider such as a $1\times N$ multi-mode interference (MMI) beam splitter to distribute the power equally [4] to all cavities. Then, for a chosen value of θ, the phase gradient can be implemented by two methods: (1) varying the length of the connection or (2) by using zero-loss resonators as all-pass filters. In the first method, the length of each waveguide is varied, for example

by meandering the path, so that the phase is tuned at the connection (see Supplementary Figure 7)(a)). In the second case, each waveguide is coupled to a zero-loss resonator. The resonators acts as all-pass filters and transmit the light perfectly. However, the transmitted light picks up a phase that is related to the resonance frequency of the resonator. This frequency, and consequently the phase, can be tuned by temperature. Therefore, by using a heater, the phase of the drive at each site can be tuned [5]. Finally, each connection is evanescently coupled to the optomechanical crystal at its corresponding position [6].

SUPPLEMENTARY REFERENCES

[1] Mingo, N., Hauser, D., Kobayashi, N., Plissonnier, M. & Shakouri, A. "Nanoparticle-in-Alloy" Approach to Efficient Thermoelectrics: Silicides in SiGe. *Nano Lett.* **9**, 711–715 (2009).

[2] Esfarjani, K., Chen, G. & Stokes, H. T. Heat transport in silicon from first-principles calculations. *Phys. Rev. B* **84**, 085204 (2011).

[3] Jeong, C., Datta, S. & Lundstrom, M. Thermal conductivity of bulk and thin-film silicon: A Landauer approach. *J. Appl. Phys.* **111**, 093708 (2012).

[4] Hosseini, A. *et al.* 1×N Multimode Interference Beam Splitter Design Techniques for On-Chip Optical Interconnections. *IEEE J. Sel. Top. Quantum Electron.* **17**, 510–515 (2011).

[5] Mittal, S., Ganeshan, S., Fan, J., Vaezi, A. & Hafezi, M. Measurement of topological invariants in a 2D photonic system. *Nat. Photonics* **10**, 180–183 (2016).

[6] Gröblacher, S., Hill, J. T., Safavi-Naeini, A. H., Chan, J. & Painter, O. Highly efficient coupling from an optical fiber to a nanoscale silicon optomechanical cavity. *Appl. Phys. Lett.* **103**, 181104 (2013).
